# Constrained acetabular liners are a viable option in second-stage re-implantation for chronic infected total hip arthroplasty with abductor or greater trochanter deficiency and large acetabular bone defects

**DOI:** 10.1186/s12891-022-05861-1

**Published:** 2022-10-14

**Authors:** Qiang Xiao, Tingxian Ling, Kai Zhou, Mingcheng Yuan, Bing Xu, Zongke Zhou

**Affiliations:** 1 Department of Orthopedics, West China School of Medicine, West China Hospital, Sichuan University, 37# Wuhou Guoxue Road, 610041 Chengdu, People’s Republic of China; 2 Department of Orthopedics, Chengdu Second People’s Hospital, Chengdu, People’s Republic of China

**Keywords:** Constrained acetabular liners, Second stage re-implantation, Abductor mechanism deficiency, Large acetabular bone defects

## Abstract

**Background:**

Abductor mechanism deficiency is a clear indication for using constrained acetabular liners (CALs), and large acetabular bone defects are considered a relative contraindication to CALs. We report the results of using CALs in special cases in which abductor or greater trochanter deficiency was accompanied by large acetabular bone defects at second-stage re-implantation for chronic infected total hip arthroplasty (THA).

**Methods:**

Between January 2010 and January 2018, 19 patients who used CALs at second-stage re-implantation and had abductor or greater trochanter deficiency and large acetabular bone defects were included in this study. We followed up with the clinical and radiological results of these patients. Complications and infection-related information were also recorded.

**Results:**

Eight patients, 4 patients, and 7 patients had Paprosky type IIB, type IIC, and type IIIA acetabular bone defects at second-stage re-implantation, respectively. The indication for using CALs was abductor deficiency in 14 patients and greater trochanter deficiency in the other 5 patients. The mean follow-up was 74.4 months (range 50–96). The mean Harris Hip Score (HHS) was 76.3 points (range 62–86) at the last follow-up. Three patients presented acetabular radiolucent lines with no progress: zone 3, zone 3 and zone 2 and 3, respectively. One patient suffered transient sciatic nerve palsy. There was no dislocation, failure of the CALs, reoperation, or recurrence of infection.

**Conclusion:**

Our results suggested that for screened patients, CALs are a viable option in second-stage re-implantation for chronic infected THA with abductor or greater trochanter deficiency and large bone defects.

## Introduction

Dislocation after primary and revision total hip arthroplasty (THA) remains one of the most troublesome complications of this procedure [1]. Two-stage revision of THA for infection is related to a high postoperative dislocation rate and the high rate is associated with gender, previous dislocation, and abductor mechanism deficiency [2]. Among these dislocation-related factors, abductor mechanism deficiency is the mainly one, as abductor or greater trochanter deficiency caused by repeated operations or infection itself is commonly seen in these cases [2]. Large-diameter heads, dual mobility (DM) acetabular components, and constrained acetabular liners (CALs) are three main implants used to prevent the dislocation of THA [3]. However, large-diameter heads showed no significant effect in terms of decreasing rates of dislocation in THAs with abductor deficiency [4]. In addition, large-diameter heads can lead to increased volumetric wear [3]. A previous study showed that DM acetabular components achieved satisfactory short- to mid-term results in the revision of THA with abductor deficiency [5]. However, long-term follow-up results of DM acetabular components used for revision THA with abductor deficiency are lacking. Moreover, the possibility of aseptic loosening, osteolysis, accelerated polyethylene wear, or intraprosthetic dislocation and the relatively high cost of DM components need to be taken into consideration [6]. One of the most common indications for using CALs is abductor mechanism deficiency causing instability of the THA [7]. The nature of CAL design underlies their disadvantages and potential failure risk: limited range of motion (ROM), impingement causing increased transmission of stress to the implant-bone interface, and thinner polyethylene and increased bearing surface areas contributing to polyethylene wear [8, 9]. For these reasons, CALs may be more suitable for patients with low activity demands [3, 10]. Furthermore, when CALs are used for THAs with large acetabular bone defects, the increased forces at the implant-bone interface affect the initial stability of acetabular components and should be of great concern [11]. Recent reviews of CALs show that the results of previous studies vary, and all these studies combine the results of CALs for hip instability caused by different mechanisms [7, 12, 13]. In addition, to the best of our knowledge, few studies have reported the results of CALs used in special cases of second-stage re-implantation for chronic infected total hip arthroplasty with abductor or greater trochanter deficiency and large bone defects. Therefore, we conducted this retrospective study to report the clinical and radiographic outcomes of CALs used for second-stage revision THAs.

## Materials and methods

This study was approved by the Ethics Committee of our institution, and all patients signed informed consent forms. We systematically searched our hospital’s joint replacement database between January 2010 and January 2018 and identified patients : (i) diagnosed with chronic periprosthetic joint infection (PJI) after THA and had undergone two-stage revision; (ii) had large acetabular bone defects (Paprosky type IIB or higher); (iii) had abductor or greater trochanter deficiency and used CALs at second-stage re-implantation. One patient was lost to follow-up, and the other 19 patients (19 hips) were included in this study. All patients’ clinical, radiographic and surgical data were available.

According to 2018 International Consensus Meeting (ICM) criteria for periprosthetic joint infection [14], a diagnosis of PJI was made if one or more of the following criteria were met: (i) microorganism in at least two cultures of synovial fluid or periprosthetic tissue; (ii) presence of a sinus tract communicating with the joint or visualizing of the prosthesis; or (iii) with an aggregate score of greater than or equal to 6 according to the scoring items. We defined greater trochanter deficiency as the greater trochanter being absent or ununited with no possibility of reconstruction. Abductor muscle quality was graded from 0 to III according to Zywiel et al. [15]: grade 0 was defined as no functional abductors; grade I was defined as marked abductor defect; grade II was defined as gross fibrosis and scarring of abductors with no abductor mass defect; and grade III was defined as abductor mass with no defects and no gross fibrosis. We primarily recorded the classification of gluteus medius and abductor deficiency of patients included in this study was grade 0-II. Large acetabular bone defects were defined as Paprosky type IIB or higher [16], and no patients in this study had pelvic discontinuity.

Before second-stage reimplantation, all patients had shown eradication of the infection according to the criteria of 2018 ICM [14]. All operations were performed by 5 senior surgeons. The same posterior approach as used in the previous surgery was used in all patients, and synovial fluid was routinely collected intraoperatively for bacterial culture prior to antibiotic administration. Intraoperative frozen section examination was performed when residual infection was suspected. If frozen sections showed more than 10 polymorphonuclear leukocytes per high-power magnification field, residual infection was considered [17, 18]. If there was definite residual infection, we performed a debridement and irrigation before second-stage re-implantation. Before implanting the revision components, we alternated use of hydrogen peroxide and povidone iodine to irrigate the joint cavity for 15 min. Abductor muscle quality, greater trochanter deficiency and bone defects were assessed intraoperatively. The use of CALs, augments, and bone allografts was decided by surgeons intraoperatively. The Pinnacle ESC Constrained Liner System (DePuy, Warsaw, IN) was used in all patients. Seventeen patients used SOLUTION femoral stems, and 2 patients used CORAIL femoral stems (DePuy, Warsaw, IN). All patients used multihole cups for multidirectional screws to supplement fixation. If possible, ischial and pubic screw fixation was used.

Postoperative antibiotics were used until the synovial fluid culture results were negative; otherwise, selective antibiotics according to the drug sensitivity test were used for 6 weeks. Partial weight-bearing with crutches began on the second postoperative day and then gradually progressed to full weight-bearing after 6 weeks according to the clinical and radiological assessment. All patients were required to avoid excessive hip abduction within the first 6 weeks and hip flexion of more than 90 degrees. All patients indicated that they could accept the limitation of hip range of motion in preoperative conversation.

Postoperative follow-up was routinely performed at 6 weeks, 3 and 6 months and then once per year. Radiological analysis was performed by 3 authors (QX, BX, and Z-K. Z), and the analysis was carried out on standard anteroposterior radiographs of the pelvis and anteroposterior and lateral radiographs of the hip. Inclination and anteversion angles were measured with the methods described by Murray [19] and Bachhal et al. [20], respectively. Both inclination and anteversion measurements were based on the ellipse of the constrained liners’ locking ring [21]. Radiolucent lines and osteolysis around the acetabular components were recorded with DeLee and Charnley zones [22]. Radiographs were also used to evaluate the presence of greater trochanter deficiency. According to McAuley et al. [23], acetabular component loosening was defined as migration of the components by more than 2 mm, screw breakage, or the presence of circumferential radiolucent lines around the components. The failure type of CALs was categorized into five categories according to Guyen et al. [9]. Clinical outcomes were assessed by the Harris Hip Score (HHS) [24]. Complications were also recorded.

### Statistical analysis

SPSS version 22 (SPSS version 22; IBM Corporation, USA) was used to perform statistical analyses. Quantitative data are presented as the mean values and ranges, and categorical data are presented as percentages.

## Results

Nineteen patients were included in this study, and the mean follow-up time was 74.4 months (range 50–96). The demographic data of the patients at the second-stage procedure are shown in detail in Table 1. There were 12 males and 7 females, and the mean age was 57.3 years (range 41–80). The average body mass index was 24.6 kg/m^2^ (range 20.9–30.0). Before the second-stage re-implantation, the affected hip had suffered an average of 2.9 prior operations (range 2–9). There was one patient (patients no.10) had 9 previous operations because he suffered several times failed debridement trying to preserve the prosthesis and experienced a failed second-stage revision at other hospital. The mean interval between one and two stages was 12.8 months (range 6–48). The pathogens at the first stage are summarized in Table 2.

**Table 1:** Demographic data of the patients at second stage

No.	Sex	Age (yr)	ASA grade	BMI (kg/m^2^)	Side	No. of prior Operations	Interval between stages (mo)	Follow-Up (mo)
1	F	51	2	25.2	Left	3	12	96
2	M	61	3	23.5	Right	2	12	92
3	M	41	2	20.9	Left	4	6	90
4	M	80	3	22.1	Right	2	9	88
5	F	51	2	30.0	Right	2	48	86
6	M	49	2	24.7	Left	3	12	81
7	M	57	2	26.7	Right	3	12	80
8	M	44	2	23.0	Right	2	6	79
9	M	58	2	24.6	Left	2	9	78
10	M	44	2	24.0	Right	9	12	74
11	F	67	3	27.0	Right	2	12	73
12	M	65	3	25.0	Left	3	12	71
13	F	48	2	24.5	Right	3	18	70
14	F	70	3	23.6	Right	2	12	69
15	F	56	2	23.3	Right	4	12	68
16	M	53	2	27.4	Right	3	9	65
17	M	75	3	22.7	Right	2	12	53
18	M	69	3	24.6	Right	2	6	51
19	F	49	2	25.5	Left	3	12	50

*ASA* American Society of Anesthesiologist, *BMI* Body mass index

**Table 2:** Microorganism of the synovial fluid culture at first stage

Microorganism identified	No. of patients
*Staphylococcus epidermidis*	10
*Staphylococcus aureus* (methicillin sensitive)	5
*Staphylococcus haemolyticus*	1
*Escherichia coli*	1
*Enterobacter cloacae*	1
Negative results	1

Among these patients, 8 (42.1%) patients had a Paprosky type IIB acetabular bone defect, 4 (21.1%) patients had a type IIC defect, and the other 7 (36.8%) patients had a type IIIA defect. The indication for using CALs was abductor mechanism deficiency caused by abductor deficiency or greater trochanter deficiency (14 patients and 5 patients, respectively) (Table 3).

**Table 3:** Preoperative clinical and radiological characteristics

Variable	Value
HHS before first-stage revision, mean (range)	22.9 (13-39)
Paprosky Classification, n (%)	
IIB	8 (42.1)
IIC	4 (21.1)
IIIA	7 (36.8)
IIIB	0
Abductor deficiency, n (%)	14 (73.7)
Grade 0	4
Grade I	6
GradeII	4
Greater trochanter deficiency, n (%)	5 (26.3)
Original implants, n (%)	
Hemiarthroplasty	1 (5.3)
THA	18 (94.7)
Original cup fixation, n (%)	
Cemented	4 (21.1)
Cementless	15 (78.9)

*THA* Total hip arthroplasty

**Fig. 1:**
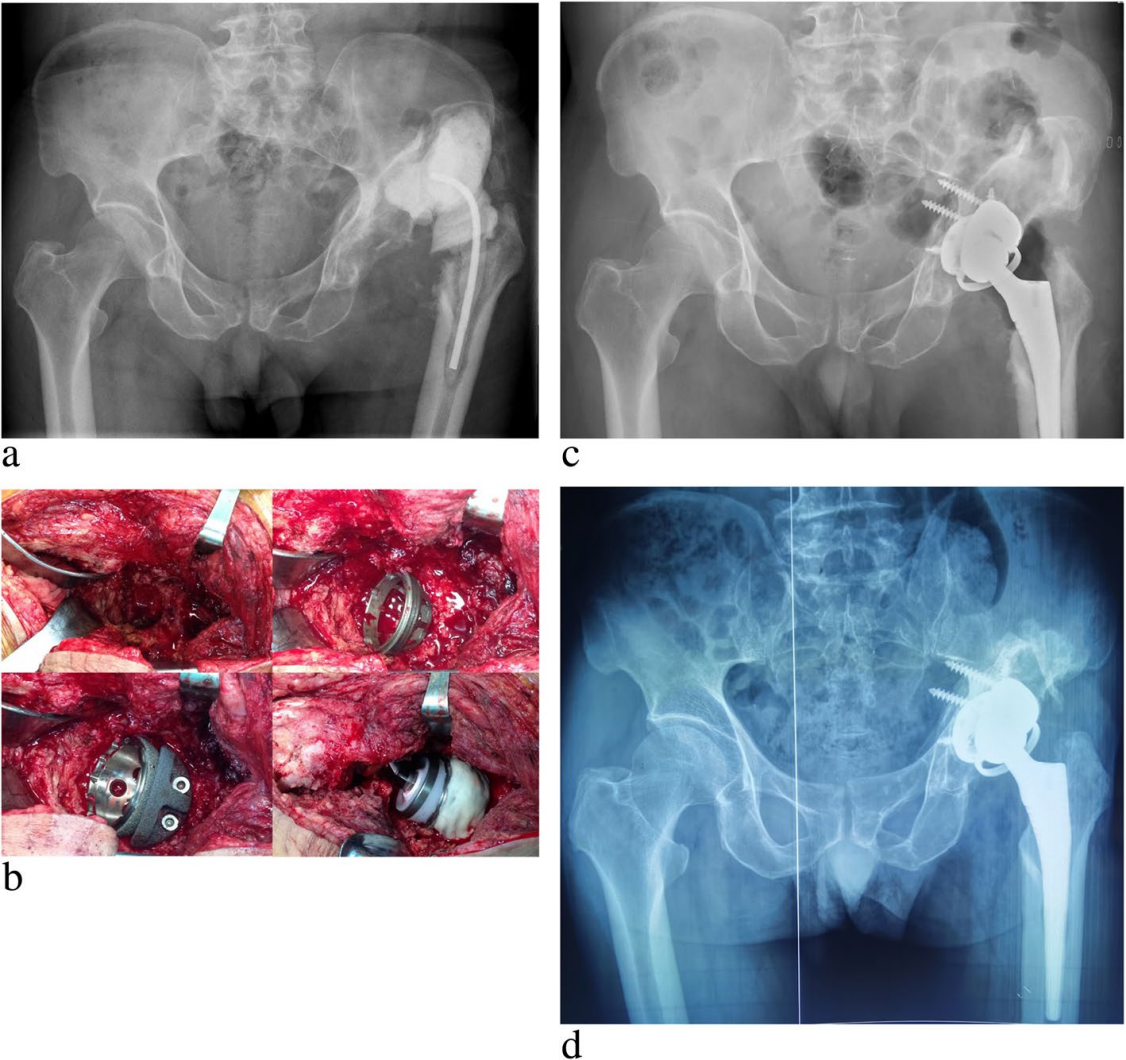
A case of a 65-year-old man (patient No. 12) with absence of gluteus medius (grade 0) and Paprosky IIIA acetabular bone defect at second-stage re-implantation, in which CAL and augment were used to reconstruct the bone defect. **a** Preoperative anteroposterior (AP) radiograph showing Paprosky IIIA acetabular bone defect and dislocation of the affected hip, **b** intraoperative finding of gluteus medius absence and a large bone defect on the top and posterior aspect of the acetabulum, **c** postoperative AP radiograph at Day 1, and **d** postoperative AP radiograph at 71 months showing acetabular radiolucent lines presenting at zone 2 and 3, and acetabular components remaining stable. HHS of the patient was 85 at the most recent follow-up

The clinical and radiological results are presented in Table 4. The mean HHS of the patients was 22.9 points (range 13–39) before first-stage revision (Table 3), which improved to 76.3 points (range 62–86) at the last follow-up. The mean diameter of the cups used in the patients was 53 mm (range 48–60). An average of 3 screws (range 2–5) was used to fix the cups. Two (10.5%) patients used one half-moon tantalum augment (Zimmer) (Fig. 1), and 5 (26.3%) patients used morselized bone graft for impaction bone grafting to reconstruct the acetabular bone defects. The mean cup inclination was 42.7° (range 32.0°-51.0°), and the mean cup anteversion was 15.2° (range 5.1°-24.1°). According to Lewinnek et al. [25], one patient’s cup inclination was outside the safe zone for the prosthesis position, and no patient’s cup anteversion was outside this zone. A 28 mm femoral head was used in 7 (36.8%) patients, while 7 (36.8%) patients received a 32 mm head, and 5 (26.3%) patients received a 36 mm head.

**Table 4:** Clinical and radiological results

Variable	Value
HHS at last follow-up, mean (range)	76.3 (62-86)
Rating, n (%)	
Excellent	0
Good	7 (36.8)
Fair	9 (47.4)
Poor	3 (15.8)
Mean cup diameter, mm (range)	53 (48-60)
No. of screws used for cup, mean (range)	3 (2-5)
Femoral head size, n (%)	
28+1.5	4 (21.1)
28+5	3 (15.8)
32+1	4 (21.1)
32+5	3 (15.8)
36+1.5	1 (5.3)
36+5	4 (21.1)
Head type, n (%)	
metal	3 (15.8)
Ceramic	16 (84.2)
Cup inclination, mean (range)	42.7° (32.0°-51.0°)
Cup anteversion, mean (range)	15.2° (5.1°-24.1°)
Patients used augment to reconstruct acetabular bone defects, n (%)	2 (10.5)
Patients used allografts to reconstruct acetabular bone defects, n (%)	5 (26.3)
Acetabular radiolucent lines, n (%)	3 (15.8)
One zone	2
Two zone	1
Three zone	0
Positive cultures at second stage, n (%)	1 (5.3)
Complications, n (%)	3 (15.8)
Fatty fluidization of the incision	2
Transient sciatic nerve palsy	1

*HHS* Harris Hip Score

After the second-stage re-implantation, one patient had the same positive synovial fluid culture result as the first stage (*Staphylococcus epidermidis*). This patient used intravenous sensitive antibiotics for 6 weeks after surgery and was free from recurrence of infection at the last follow-up. One patient suffered transient sciatic nerve palsy and fully recovered at 3 months after surgery. Two other patients had fatty fluidization of the incision, and the wounds healed after several dressing changes. Two patients presented acetabular radiolucent lines in one zone (zone 3) and 1 patient presented in two zones (zone 2 and 3) with no progress (Fig. 1), and the implants of all patients remained stable at the last follow-up. Furthermore, no dislocation or failure of the CALs occurred.

## Discussion

Currently, two-stage revision with a spacer is the most commonly used modality for chronic PJI after THA; however, as a result of multiple prior operations and infections, some patients experience abductor or greater trochanter deficiency at second-stage re-implantation and are faced with a high risk of dislocation [2]. Moreover, as spacer placement causes progressive bone loss and the removal of the original prosthesis can lead to iatrogenic bone loss, acetabular bone defects are not uncommon at second-stage re-implantation [26]. Both abductor deficiency and greater trochanter deficiency result in abductor mechanism deficiency and are clear indications for using CALs [7]. However, some authors suggest that CALs are generally avoided in patients with large acetabular bone defects due to the fear of loosening at the bone-implant interface [7, 13]. In contrast, a 10-year follow-up study of CALs, including cases with large acetabular bone defects, showed no failure at the bone-implant interface [21]. To the best of our knowledge, there are no studies reporting CAL use in cases in which abductor or greater trochanter deficiency accompanies large bone defects, much less in cases of second-stage re-implantation. Our study, despite its small sample size, showed that CALs used in such situations at second-stage re-implantation could achieve satisfactory mid-term results.

Recent reviews have summarized the literature about CALs used in revision THA, and the results were as follows: in the short-term (0–5 years), the dislocation rate was 0–29%, the acetabular component survival rate was 71-97.2%, and the cup loosening rate was 0-3.8%; in the mid-term (5–10 years), the dislocation rate was 0.8–21%, the acetabular component survival rate was 70.4–90.8%, and the cup loosening rate was 0-4.3%; and in the long-term (> 10 years), the dislocation rate was 7.1–18.6%, the acetabular component survival rate was 54.7–85%, and the cup loosening rate was 0-8.3% [12, 13]. Our series with mid-term follow-up showed that there was no dislocation or acetabular component failure. We think there are several factors contributing to these good results. First and foremost, selecting the right patients is crucial. Previous studies have mentioned that CALs should be used in patients with low activity demand [3]. The design of CALs allows for a reduced ROM, so excessive motion can lead to impingement, which can cause direct damage to the locking ring and transform stress to the bone-implant interface, contributing to acetabular loosening. Moreover, the average wear rate of CALs was one-third greater than that of primary THA [9, 27]. Therefore, before surgery, it is necessary to evaluate the postoperative hip function of the patients and know the patients’ expectations about the revision surgery. Although the last follow-up mean HHS of the patients in our study was fair, all patients had a high level of satisfaction. Second, the position of the implants is important. When the acetabular components are malpositioned, standard functional hip movement can lead to impingement and then result in failure of the CALs and cup loosening [7]. In our study, there was only one patient whose cup inclination was slightly greater than the safe zone described by Lewinnek et al. [25]. Third, using screws to supplement fixation for acetabular components is necessary. As the impingement of CALs can transform stress to the bone-implant interface, the initial stability is important for a new implant cup [9, 21]. The follow-up of a large series of 148 revision THAs by Bedard et al. showed that newly implanted tantalum acetabular components fixed with a large number of screws can resist the bone-implant interface stress from the impingement of CALs, with no failure at the bone-acetabular implant interface at 10 years [21]. In our study, we used a mean of 3 screws (range 2–5) to supplement fixation for the cup. At the same time, 2 patients used augment, and 5 patients used allografts to reconstruct the bone defect. In this study, we aimed to achieve at least 80% contact between the components and the host bone. Fourth, postoperative patient management also plays an important role. Although the product data sheet of the acetabular system (Pinnacle ESC, DePuy, Warsaw, IN) used in our study claims that 28-mm, 32-mm, and 36-mm heads could achieve a ROM of 92°, 96°, and 104°, respectively, all the patients were required to restrict hip flexion to 90° and avoid excessive abduction.

The use of DM acetabular components has gradually become one of the leading ways to manage hip instability in the last decade. The main mechanisms contributing to the favorable stability of DM acetabular components are as follows: an increased femoral head-to-neck ratio makes the jump distance of the femoral head increase; and two articulations allow a greater ROM before impingement [28]. A systematic review conducted by Darrith et al., including 3008 DM revision THAs, showed that the dislocation rate was 2.2% and the survival rate was 96.6% at a mean follow-up of 5.4 years (range 2–8) [28]. These results are superior to the midterm follow-up results of CALs mentioned before. Additionally, Klemt et al. recently reported using DM in 42 revision THAs with abductor deficiency caused by adverse local tissue reaction, and the average 4-year follow-up demonstrated that there was no dislocation, and the implant survival rate at the 3-year follow-up was 87% [5]. Overall, the current studies show that DM seems to have a promising application in dealing with hip instability. Some authors have even suggested that DM can replace CALs to manage some complex revision THAs with a high risk of dislocation, such as abductor deficiency [29]. However, long-term follow-up studies of DM revision THAs are required to eliminate concerns about aseptic loosening, osteolysis, accelerated polyethylene wear, and intraprosthetic dislocation [6]. In addition, it is necessary to consider the cost of DM compared with CALs. A large head is also an option for hip instability, but in patients with abductor or greater trochanter deficiency, it may not be effective [4, 10].

There are some limitations to our study. First, this was a retrospective study with no comparison group. Second, the sample was small, and the follow-up time was mid-term, which decreased the level of evidence. However, the type of patient included in this study is relatively uncommon, which is evident from the lack of similar previous studies. Third, the operations were performed by 5 senior surgeons in our center. The use of different surgical techniques may affect the outcomes. However, there was no failure case, which may reflect that using CALs in second-stage re-implantation in patients with abductor or greater trochanter deficiency and large bone defects is a viable option for different surgeons.

In conclusion, our results suggested that CALs were a viable option in second-stage re-implantation for chronic infected THA with abductor or greater trochanter deficiency and large bone defects. It is worth mentioning that deliberate patient selection may have contributed to our good results.

## Data Availability

The datasets supporting the conclusions of this article are included within the article. The raw data can be requested from the corresponding author on reasonable request.

## Abbreviations

CALs: Constrained acetabular liners
HHS: Harris hip score
THA: Total hip arthroplasty
ROM: Range of motion
PJI: Periprosthetic joint infection
DM: Dual mobility

## Declarations

All methods were performed according to the appropriate guidelines and regulations.
